# The Scientific Basis of Medicine

**Published:** 1897-11

**Authors:** 


### BOOK NOTICES.

THE SCIENTIFIC BASIS OF MEDICINE. By I. W. Heysinger, M. A., M. D. Philadelphia: Bœricke & Tafel, 1011 Arch Street. 1897. Price, 50 cents net. By mail, 55 cents.

This little book is designed to set forth the advantage of Homœopathy as a method of medical treatment, and to establish it upon a scientific basis.

The author believes that by reason of prosperity and success we are gradually corrupting the practice of Homœopathy, and are in imminent danger of losing all that we have gained.

" Eternal vigilance," he says, " is the price of liberty," and with the attainment of liberty " comes the carelessness of self-content, and then follow the vagaries of the mystic, with the schemes of the individual self-seeker—what is everybody's business is nobody's business, and finally the sacred cause is imperiled, and all its glorious fruits are lost." Hence it is necessary to take our bearings and find out, as the Western Congressman said, " Where we are at." This little book is therefore put out with a view of bringing the profession back to the standard right line of pure principle.

We are not satisfied that the author has demonstrated this right line, and accomplished his object.

His comparisons with the old school of medicine, and exposition of its weaknesses are not new enough to be very stimulating, and his attacks upon what may be called the vices of practice in the new school are not always close enough to the fact to make them effective. Thus on page 26, in his attack upon the keynote system, while what he says is a well-deserved rebuke to those who *abuse* the keynote system, he fails to tell what the real value of the keynote is, what service it has rendered, and what a loss it would be if expunged from the materia medica. He states the keynote system thus : " Taking three individual symptoms in any case presented, it was argued that like three points taken in geometry a therapeutic circle could be drawn which should include the whole, and thus the curative remedy could be marched up, as it were a corporal and a file of dragoons. It was not necessary to know anything about anything," etc.

Now we think that the foregoing is not a correct statement of the " keynote," which, we may add, is not a "theory " at all.

We think that a better statement of the keynote is that it is a certain symptom which occurs under a certain remedy in its provings so often, and has been verified in clinical practice so frequently that it stands out under that remedy, and is more identified with that remedy than with any other under which it may occur.

A *homœopathic* practitioner seeks the totality of the symptoms. When he fails to get the totality, he determines the remedy by the greatest number he can find. If two or more remedies confront him, having each a large number of symptoms, of probably inferior importance, then the keynote will decide the choice between them. If no remedy can be found which has any considerable number of the patient's symptoms, and there should turn up a keynote, he avails himself of that and not infrequently cures his case. The resorting to the keynote under this last condition is certainly better than letting the patient suffer or resorting to a mustard plaster or a fifteen-grain dose of Quinine or a hypodermic injection of Morphine, as do those who call themselves liberals, or "the new school of Homœopathy."

The author speaks of selecting "three individual symptoms," as if these symptoms could be chosen at random. This would, of course, lead to certain failure. When only three symptoms are chosen, they must be the most characteristic symptoms of the patient and at the same time the most characteristic or keynote symptoms of the remedy; and what is more, this method is to be pursued only when there is an impossibility of getting the totality of the symptoms. Hence, the author unintentionally gives a false impression about the keynote to those who are imperfectly acquainted with the homœopathic principle. This is to be regretted in a book that aims to bring the profession back to homœopathic bearings.

With regard to the plum pudding incident, on page 28, it may be suggested that if the plum pudding caused cramps, then there must have been in its composition some "sick-making" element worthy of investigation as a drug. Eminent chemists have not felt it beneath their dignity to investigate *custard* puddings, and their labors were rewarded by the discovery of poisonous ptomaines. That the plum pudding incident should appear ludicrous, while the *custard* pudding story should be invested with scientific dignity, is all a matter of illumination, of the kind of light in which you choose to put each one. The tin-cup story is, of course, ludicrous, but we submit that it is not enough to overthrow the whole homœopathic principle.

Many fine things are said concerning Hahnemann and his doctrines, as, for example, on pages 36 to 41, or pages 52 to 64, and elsewhere.

The sarcasms on the fluxion or bottle-washed potencies are caustic and amusing.

The remarks upon the single remedy are disappointing and calculated to induce the younger generation of practitioners to diverge more widely from the true homœopathic bearings instead of bringing them more closely to it.

The author asks why the materia medica should ever have been enlarged. We think this question needless, and brings its own answer by a simple survey of the field of medical phenomena. Hahnemann laid no limitations in that direction. He merely pointed out how to make additions that would be of value. We think it a great oversight to say of totality that "it is totality or nothing." When a drug is selected that has the exact totality of the symptoms of the patient, there is almost no appreciable interval of time between its administration and the beginning of curative action. Its action is almost instantaneous. We commonly hear it said of it under such circumstances, "its action was a miracle." A perfect cloud of witnesses can give this testimony.

When a drug has *no* similarity to the case, its effects are nothing, or it produces disastrous results according to circumstances. Between these two extremes there are all degrees of approach to similarity, and hence we find remedies producing palliation of symptoms, and so a sequence of many remedies may be needed each only more or less remotely similar, each one producing *some* salutary effect until the patient may be said to take a zigzag course, so to speak, back to a state of health.

The argument for alternation "at proper intervals" is fallacious. The author well knows that the phrase "proper intervals" means with the great horde of practitioners a mere arbitrary and capricious setting of periods of time between doses. These caprices soon become of the most wanton order, utterly indifferent to the relationships of the drugs to each other or to the patient, and are resorted to in order to shirk the labor of study. The prescribing of two drugs in alternation is not only a defiance of the reasonableness of the homœopathic principle, but it leads inevitably to the use of schemes of rotation of three, four, five, and even six drugs at a time. There are many authentic instances reported constantly of just this kind of prescribing. This is polypharmacy with a vengeance.

Thus the minds of the professed homœopathists are made familiar with polypharmacy, and its folly is no longer apparent to them. The druggists are meeting this mode of thought half way, and putting within their reach tablets and other preparations containing several drugs mixed together after the manner of the dominant school, and impudently claiming these preparations "modern" Homœopathy, "The New School of Homœopathy," and "Progressive Homœopathy" when any one not stone blind to facts knows it to be the most atrocious empiricism.

Empiricism and polypharmacy always have been the curse of the old school of medicine, and the druggists were its tyrants.

Hahnemann when he came forward with the law of cure and the principle for its application, at one blow overthrew these monsters and emancipated his followers from their thrall.

Now come these same monsters in more insidious guise, seeking to re-establish their old supremacy, and our author, notwithstanding all his cleverness and wit, and his otherwise fine presentation of Hahnemann's discoveries, is assisting them to regain their prestige and power.

Here is a glaring failure to achieve the object of the book, the return of the school to the true bearings.

The author asserts that a drug cannot be homœopathic to a case unless *the case* contains every symptom that is found under the drug! Thus, if a case have five hundred symptoms, every one of which is found under a certain drug, and meanwhile the drug has in its pathogenesis one thousand symptoms, then the drug is not homœopathic to the case because *the case* does not contain all these thousand symptoms!! He further avers that it is not homœopathic if the drug contain even one more symptom than the case!!! This is an astounding statement for a professed homœopathist! If so, no physician who uses the single remedy ever gives a simillimum; consequently he never cures a case, and all the cures reported are falsehoods. There is no escape from this conclusion.

Those who alternate do no better, according to the view laid down by the author, for they do not cover the symptoms either. They give drugs at random and resort to any device to save the labor of study of the materia medica. That is what alternation really means, and our author appears to be apologizing for it.

It is to be distinctly understood that the reviewer in no wise overlooks the merits of the book. Some of these have been mentioned. If there were no good points in it it would not have received the compliment of so long a review.

